# Bioaccessibility and Genoprotective Effect of Melanoidins Obtained from Common and Soft Bread Crusts: Relationship between Melanoidins and Their Bioactivity

**DOI:** 10.3390/foods12173193

**Published:** 2023-08-24

**Authors:** Virginia Temiño, Gisela Gerardi, Monica Cavia-Saiz, Noelia Diaz-Morales, Pilar Muñiz, Gonzalo Salazar

**Affiliations:** Department of Biotechnology and Food Science, Faculty of Sciences, University of Burgos, Plaza Misael Bañuelos, 09001 Burgos, Spain; vtemino@ubu.es (V.T.); mggeradi@ubu.es (G.G.); monicacs@ubu.es (M.C.-S.); gsalazar@ubu.es (G.S.)

**Keywords:** melanoidins, bioaccessible fractions, membrane permeability, transepithelial transport, genoprotective effect

## Abstract

Bread crust constitutes an important by-product of the bakery industry, and its utilization for the isolation of melanoidins to be used as a functional ingredient can enhance its added value and contribute to health. The aim of this study was to evaluate the bioaccessibility, bioactivity, and genoprotective effect of melanoidins derived from bread crust. Bioaccessibility was assessed in gastric, intestinal digestion, and colonic fermentation fractions. The results revealed a relationship between bioaccessible melanoidins and their type (common or soft bread). No cytotoxicity effects were observed for bioaccessible fractions, as assessed by MTT and RTA methods, and they did not affect the distribution of E-cadherin in Caco-2 cells, confirming their ability to maintain membrane integrity. Furthermore, our study demonstrated that the gastrointestinal and colonic fermentation fractions successfully transported across the intestinal barrier, without affecting cell permeability, and showed antioxidant activity on the basolateral side of the cell monolayer. Remarkably, both fractions displayed a significant genoprotective effect in Caco-2 cells. Our findings provide crucial insights into the relationship between the melanoidins and their bioactivity and genoprotective effect. These results demonstrated the potential of bioaccessible melanoidins as valuable bioactive compounds for the development of functional foods, without showing toxic effects on gastrointestinal cells.

## 1. Introduction

Bread crust constitutes an important by-product of the bakery industry produced during the manufacturing or distribution stages [1]. Therefore, the revaluation of bakery industry by-products by obtaining compounds with high bioactive capacities from them, such as melanoidins, can contribute to reduce environmental contaminants and add value to them.

Melanoidins are high-molecular-weight, brown-colored compounds formed during the Maillard reaction, which occurs when sugars react with amino acids [2]. An increasing number of studies [3,4,5] have shown that melanoidins are associated with several beneficial biological effects, including antioxidant, antimutagenic, antihypertensive, and prebiotic properties. The health effects of melanoidins depend on their bioaccessibility, which refers to the extent to which these compounds can be released from food matrices during digestion and become available for absorption by the body. Although melanoidins are generally considered to have low bioaccessibility, some studies have suggested that the bioaccessibility is dependent on the food processing methods, such as roasting or cooking, which can break down the complex structures of these compounds and increase their solubility [6,7]. In general, the process of melanoidin digestion and colonic fermentation results in bioaccessible fractions that remove soluble compounds [8,9].

Bread crust is an important source of melanoidins and its content is influenced by various factors, such as bread formulation, baking time, and temperature. These factors affect the bioaccessibility and health properties of melanoidins [4,10,11,12]. To date, the bioaccessibility of melanoidins from common and soft bread crust has not been extensively investigated. For this reason, its study is interesting to evaluate the potential protective effect of melanoidins against dysfunction of the intestinal barrier in order to prevent systemic exposure to food toxicants and pathogens.

This study aims to characterize the bioaccessible fractions of melanoidins obtained from crusts of both soft and common bread. An in vitro digestion procedure that mimics the physiochemical changes that occur during digestion in the stomach, small intestine, and colon was carried out. The characterization and bioactivity of the digestion fractions were assessed using both chemical assays and cultured differentiated Caco-2 cells.

## 2. Materials and Methods

### 2.1. Materials and Chemicals

We used Pronase E (EC 3.4.24.4); 2,2′-azino-bis(3-ethylbenzothiazoline-6-sulfonic acid) diammonium salt (ABTS); potassium persulfate (K_2_S_2_O_8_); 6-hydroxy-2,5,7,8-tetramethylchromane-2-carboxylic acid (TROLOX); methanol (CH_3_OH); iron(II) sulfate heptahydrate (FeSO_4_ · 7H_2_O); α-calcium chloride (CaCl_2_); potassium chloride (KCl); sodium bicarbonate (NaHCO_3_); sodium phosphate monobasic (NaH_2_PO_4_); sodium phosphate dibasic (Na_2_HPO_4_); monopotassium phosphate (KH_2_PO_4_); magnesium chloride hexahydrate (MgCl_2_ · 6H_2_O); sodium chloride (NaCl); hydrochloric acid (HCl); iron (III) chloride hexahydrate (FeCl_3_ · 6H_2_O); and ammonium carbonate ((NH_4_)2CO_3_). Enzymes used in enzymatic digestion: a-amylase (EC 3.2.1.1), amyloglucosidase (E.C 3.2.1.3), lipase (EC 3.1.1.3.), and pepsin (EC 3.4.23.1.). 2′,7′-dichlorofluorescein diacetate (DCFH-DA), Bovine Serum Albumin, Fetal Bovine Serum, 200 mM L-Glutamine solution, Eagle’s Minimum essential medium (MEM), 100 X MEM non-essential amino acid solution, p-formaldehyde, and phosphate-buffered saline 1X (PBS) were purchased from Sigma-Aldrich, Co. (St. Louis, MO, USA). Sodium acetate (C_2_H_3_NaO_2_), tris(hydroxymethyl)aminomethane hydrochloride (C_4_H_11_NO_3_·HCl, TRIS); trichloroacetic acid (C_2_HCl_3_O_2_); acetic acid (CH_3_COOH); and 2,4,6-tri(2-pyridyl)-s-triazine (TPTZ) were obtained from Thermo Fisher Scientific (Waltham, MA, USA).

### 2.2. Bakery Samples

Samples of soft bread crusts were obtained from Cerealto-Siro Company as by-products, and common bread was elaborated in the Food Technology Department of University of Burgos (Spain). Crust samples were ground in a mill and sieved to a particle size < 1 mm in a wire mesh sieve (CISA, Barcelona, Spain).

### 2.3. Isolation of Melanoidins

Melanoidins from the two-bakery product (common and soft bread) were extracted by in vitro digestion with Pronase E according to the methods described by [13] with slight modifications [14]. Briefly, 125 g of crust powder was enzymatically hydrolyzed with 750 mL of a 400 U/mL solution of pronase E in 20 mM Tris-HCl buffer (pH 8.0), for 72 h at 37 °C with continuous agitation in digestion jars of a “Daisy” incubator (ANKOM Technology Corporation, New York, NY, USA). After digestion, samples were mixed with 15 mL of 40% trichloroacetic acid solution (*w*/*v*) and centrifuged (10,800 rpm at 4 °C for 10 min). The soluble fraction was subjected to ultrafiltration to obtain the melanoidin solution. Ultrafiltration experiments were performed under the dead-end configuration using a stirred-cell with the flat-sheet polyethersulfone ultrafiltration membrane (Trisep Flat Sheet Membrane UF10, Sterlitech Corporation, Washington WA, USA), and a 10 kDa nominal molecular mass cutoff was used. Melanoidins (the retained fraction) were freeze-dried (FreeZone 12 L Console Freeze Dry System with drying chamber, Labconco, MO, USA).

### 2.4. Bioaccessible Melanoidin Fractions: In Vitro Digestion and Colonic Fermentation

The in vitro digestion of the samples was carried out following the procedure described by Minekus et al. [15] with some modifications. Initially, 1 g of the sample was mixed with 20 mL of simulated fluid solution (SFS) at pH 7, composed of α-amylase (75 U/mL) and CaCl2 (0.3 M). The mixture was stirred for 2 min at 37 °C. To mimic the gastric phase, the samples were incubated with pepsin (500 U/mL) at pH 1.5 for 2 h at 37 °C (G sample). After that, in the intestinal phase, the pH was adjusted to 7.5 with 1 M NaHCO_3_, incubated with an enzyme solution of pancreatin (100 U/mL) and bile salts (10 mM) for 2 h at 37 °C, and centrifuged at 6000× *g* for 10 min, and the supernatant was collected (GI sample). The solid residue obtained by centrifugation contained the undigested compounds of the small intestine that may reach the large intestine, which was the substrate for the action of colonic microbiota. The result of colonic fermentation was separated by centrifugation to collect the supernatant, obtaining the potential bioaccessible colonic fermentation fraction (F). Three replicates were carried out for each fraction. Negative controls (without melanoidin sample) were also prepared. All isolated fractions from the simulated digestive procedure were freeze-dried, weighed, and stored at −20 °C until analysis. The recovery of the digested fractions (G, GI, and F) is expressed as a percentage (% *w*/*w*) relative to the initial quantity of each fraction.

### 2.5. Absorbance of Melanoidins

The absorbances at 345 nm and 420 nm were evaluated as an indicator of the degree of browning using a spectrophotometer (U-2000 Hitachi, Ltd., Hubbardston, MA, USA) and 1 cm-path length cuvettes, following the procedure described by Gonzalez-Mateo et al. [13]. The melanoidin extracts and fractions were dissolved in milli-Q water.

### 2.6. Determination of Total Antioxidant Capacity (TAC)

Two different methods (Q-ABTS and Q-FRAP assays) were used to evaluate the antioxidant capacity of the melanoidins following the procedure described by Del Pino et al. [16]. All the experimental measurements were performed using the Synergy 2 BioTek Microplate Reader (BioTek, Winooski, VT, USA).

Q-ABTS assay—ABTS radical (2,2′-Azino-Bis-3-Ethylbenzothiazoline-6-Sulfonic acid) was generated through a 1:1 mixture of a 7 mM ABTS solution and 2.45 mM potassium persulfate. Subsequently, this ABTS^+^ working solution was combined with melanoidins (10 mg/mL). After 30 min of incubation in darkness with continuous stirring, the spectrophotometric absorbance of the samples was measured at 734 nm. To establish a calibration curve, various quantities of Trolox were employed. The outcomes are expressed as micrograms (µg) of Trolox equivalents per milligram (mg) of melanoidins.

Q-FRAP assay—The FRAP (ferric reducing power) reactive solution was freshly prepared by combining 10 mM TPTZ and 20 mM FeCl_3_ · 6-H_2_O within a 0.3 M sodium acetate buffer at a pH of 3.6, maintaining a ratio of 1:1:10 (*v*/*v*/*v*). This solution was then further diluted at a ratio of 10:1 (*v*/*v*). Subsequently, 6 mL of the FRAP solution was introduced to bioaccessible melanoidins (10 mg/mL) and allowed to incubate for 30 min at 37 °C with continuous stirring. The spectrophotometric absorbance at 593 nm was measured, and the results are quantified as micrograms (µg) of iron (II) equivalents per milligram (mg) of melanoidins. This quantification was achieved using a linear calibration established with varying quantities of FeSO_4_.

### 2.7. Determination of Radical Scavenger Activity

To assay the radical scavenger capacity of the melanoidins, the procedure described by Del Pino et al. [16] was followed. All measurements were performed using the Synergy 2 BioTek Microplate Reader (BioTek, Winooski, VT, USA).

Q-SRSC assay (superoxide radical scavenger activity)—Briefly, 5 mg of the products was mixed with 1.5 mL of 78 mM NADH, 50 mM NBT, and 10 mM PMS in 16 mM Tris-HCl (pH 8.0). The mixture was stirred and incubated for 30 min, and the absorbance (A) of the melanoidin samples at 560 nm was measured. Control samples (samples and buffer) and the oxidized control (without samples) were also prepared. The results are expressed as oxidation inhibition percentages with respect to the oxidized control.

Q-HRSC assay (hydroxyl radical scavenger activity)—5 mg of the melanoidin samples was weighed and mixed with 1 mL of 1 mM deoxyribose, 0.1 mM C_6_H_8_O_6_, 1 mM H_2_O_2_, 0.1 mM FeCl3, and 0.1 mM EDTA in 5 mM phosphate buffer (pH 7.4). Control samples (samples and buffer) and the oxidized control (without samples) were also prepared. The samples were incubated at 37 °C for 60 min with continuous stirring. After that, 1.5 mL of TCA (28% *w*/*v*) and 1 mL of TBA (1% *w*/*v*) were added and were heated at 100 °C for 15 min. The absorbance was recorded at 532 nm and the results are expressed as oxidation inhibition percentages with respect to the oxidized control.

### 2.8. Determination of Metal Chelating Capacity

The assessment of the metal chelating capacity of the bioaccessible melanoidin extract was carried out using a modified version of the method outlined by Carter et al. [17]. In this procedure, 25 μL of the sample extract was combined with 150 μL of Milli-Q water. Following this, 25 μL of 0.2 mmol/L FeCl_3_ was introduced and mixed into the solution. After a 30 s incubation at room temperature, 50 μL of a 1 mmol/L ferrozine solution was added. For quantification, the absorbance was measured at 562 nm using a microplate spectrophotometer reader (Synergy 2 BioTek Microplate). The chelation capacity of Fe^2+^ was calculated using the formula, (A0 − A1)/A0 × 100, where A0 represents the absorbance of the control sample without the presence of the tested extract, and A1 is the absorbance when the sample extract is included. This calculation allows for the determination of the percentage chelation capacity. All the samples were assayed by triplicate.

### 2.9. Cell Culture and Exposure Conditions

Human colon adenocarcinoma Caco-2 (HTB 37™, ATCC, LGC Barcelona, Spain, 32 passage number), obtained from American type culture Collection (ATCC), was cultured at 37 °C and 5% CO_2_ in Eagle’s minimum essential medium (MEM) supplemented with 20% (*v*/*v*) heat-inactivated fetal bovine serum, 1% (*v*/*v*) non-essential amino acids and 1% (*v*/*v*) L-glutamine, 2 mM L-glutamine, 100 U/mL of penicillin, 100 μg/mL of streptomycin, 1% (*v*/*v*) non-essential amino acids, and 0.5 μg/mL of amphotericin B.

#### 2.9.1. Cytotoxicity Study

MTT assay—To assess cell viability, the MTT method was employed. Cells were seeded at a density of 1 × 10^4^ cells per well in 96-well plates. Subsequently, they were incubated with the bioaccessible fractions at different concentrations (25, 50, and 250 µg/mL) for 24 h and 40 µL of MTT (0.5 mg/mL) was added and incubated at 37 °C for 2 h. The medium was removed and 100 µL of DMSO was added. The absorbance at 570 nm was measured. The results are expressed as percentage of cell viability with respect to the untreated control.

Real-Time Cell Analysis (RTA)—Cytotoxicity assay was performed using the iCELLigence real-time cell analysis technology (RTCA) (San Diego, CA USA) [18]. Caco-2 cells were seeded on L8 E-Plates (Agilent, Madrid, Spain) at a density of 1 × 10^4^ cells/well and allowed to reach confluence. Subsequently, the cells were treated with 100 μg/mL of bioaccessible (GI and F) fractions in MEM for 4 h. Changes to the impedance were continuously monitored every 5 min using the RTCA system. Cell sensor impedance is expressed as an arbitrary unit called the Cell Index. The plot shows normalized data, and curves were plotted with control wells set as the baseline. All experiments were repeated three times.

#### 2.9.2. Morphological Evaluation of the Caco-2 Cells by Immunofluorescence

Caco-2 cells were seeded at a density of 1 × 105 cells/mL in 6-well plates containing glass coverslips and grown in completed media. After twenty-one days of culture, differentiated Caco-2 cells were pretreated with 100 μg/mL of bioaccessible melanoidins fractions (GI and F) for 24 h. After fixing with 4% p-formaldehyde, the cells were permeabilized with 0.2% Tween and incubated with the corresponding staining: CytoPainter Phalloidin iFluor 488 Reagent (Abcam, Madrid, Spain) and Rb mAb to E-cadherin (EP7004) Alexa Fluor 594 (Abcam). Then, the cells were mounted with Fluoroshield mounting medium (Abcam). Images were obtained using a Leica CTR6000 microscope and LAS AF Software (Leica Microsystems, Wetzlar, Germany). The image analysis was realized using ImageJ Software (Fiji ImageJ 1.52b NIH USA).

#### 2.9.3. Genoprotective Effect: Comet Assay

Comet assays were performed following the procedure described by Del Pino et al. [19]. Caco-2 cells were cultured at a density of 2 × 10^5^ cells/well for 24 h. Subsequently, the cells were treated with 0.1 mg/mL of GI and F bioaccessible fractions of common and soft bread melanoidins dissolved in DMSO, and 15 μL of catalase (100 U/mL) for 24 h at 37 °C and 5% CO_2_. The oxidation was induced with H_2_O_2_ 0.05 mM, diluted in MEM. After that, the cell suspensions were collected and resuspended in preheated 1% low-melting-point agarose. This mixture was added to frosted microscope slides precoated with 1% normal-melting-point agarose. The slides were then immersed in a cold lysing solution (2.5 M NaCl, 100 mM EDTA, 100 mM Tris, 1% sodium-lauryl-sarcosineate, 1% Triton X-100, 10% DMSO, pH 10) overnight at 4 °C. Next, the microscope slides were placed in an electrophoresis tank and the DNA allowed to uncoil for 40 min in alkaline electrophoresis buffer (1 mM EDTA, 0.3 N NaOH, pH 10). Electrophoresis was conducted at 4 °C, 25 V, 500 mA, and 150 W. The slides were subsequently neutralized with Tris buffer (0.4 M, pH 7.5) and stained with ethidium bromide, observed using a fluorescent Leica CTR6000 microscope and LAS AF Software (Leica Microsystems, Wetzlar, Germany), and then photographed with a digital camera. The photographs were analyzed using the CometScoreTM program. For each analysis, 30 individual cells were randomly selected and their tail length evaluated (DNA in the tail).

#### 2.9.4. Transepithelial Transport Studies

Cells were seeded at a density of 100,000 cells/cm^2^ in Corning™ Transwell™ (Sigma-Aldrich, Madrid, Spain) 6-multiwell plates (12 mm Ø, 0.4 µm pore size) on the apical surface and incubated for 21 days to establish differentiation. The medium was changed every two days for 3 weeks. The transepithelial electrical resistance (TEER) between apical and basolateral compartments was evaluated with a Milli-cellERS volt-ohmmeter (Millipore, MA, USA). TEER values are recorded as Ω.cm^2^. Caco-2 monolayer was treated with gastrointestinal and colonic fractions of CBM and SBM for 4 h at 37 °C, 5% CO_2_, and 95% RH. Monolayer integrity was evaluated using 100 M of lucifer, and the amount transported to the basolateral side was evaluated through quantification with a luminometer at Ex/Em = 425 nm/350 nm. The TEER values were measured before and after the transport experiments to ensure the integrity of the monolayers and the relative TEER values were determined according to the equation:

Relative epithelial resistance (%) = (TEER value after treatment/TEER value before treatment) × 100.

The transport of melanoidins from the apical to the basolateral side of the transwell system was assessed by measuring the antioxidant capacity assayed using the FRAP method. All the experimental measurements were performed using the Synergy 2 BioTek Microplate Reader (BioTek, Winooski, VT, USA). The results obtained are expressed as epithelial transport (%) = ([final sample − control sample (untreated)]/initial sample) × 100.

### 2.10. Statistical Analysis

All data are expressed as the mean ± standard deviation (SD) of at least three independent measurements for each of the three replicate samples. Statistical analysis was performed using Statgraphics^®^ Centruion XVI, version 16.2.04 (Statpoint Technologies, Inc., Warranton, Virginia, VA, USA). Analysis of variance (ANOVA) using Fisher’s Least Significant Difference was performed to detect significant differences between the data (*p* < 0.05).

## 3. Results and Discussion

Despite bread being a primary source of melanoidin consumption, limited attention has been given to studying melanoidins in bread and their bioaccessibility. The significance of melanoidins studies lies in their widespread presence in foods and their potential use as functional ingredients, offering various benefits to human health [3,9]. To investigate the influence of the gastrointestinal digestion process on the bioavailability of melanoidins from bread crust, as well as their antioxidant and chelating activity, we evaluated the bioaccessible samples obtained through “in vitro” gastric (G), gastrointestinal (GI) digestion, and colonic fermentation (F).

The measurement of the melanoidin bioaccessibility provides valuable information for the evaluation of their biological efficacy in food products [20]. The yield values of gastrointestinal bioaccessible extract of common bread (CBM) and soft bread melanoidins (SBM) were 14.6 ± 3.3 g/100 g common bread crust and 14.03 ± 1.92 g/100 g soft bread crust. Furthermore, the results showed that the bioaccessibility of melanoidins varied through the simulated gastrointestinal tract. In this regard, the bioaccessibility of the colonic fractions was 1.21 ± 0.44 g/100 g common bread crust and 0.88 ± 0.35 g/100 g soft bread crust. These values are similar to those obtained by de Cosio et al. (2020), where it was observed that coffee melanoidins showed their highest bioaccessibility from the mouth to the small intestine, but their colonic fraction bioaccessibility was lower [21,22]. Although these results allow the estimation of bioaccessible soluble compound content across the digestion fractions, they do not serve as indicators of the content of the melanoidins, due to the fact that they are not pure fractions. Therefore, it is necessary to evaluate more specifically the characteristics of melanoidins in the bioaccessible fractions, considering their chemical, biological, and bioactive properties.

The characteristics of bioaccessible melanoidins including absorbance index, total antioxidant capacity, radical scavenger capacity, and chelating capacity are reported in Table 1. The formation of different melanoidins is associated with the changes in absorbance at 345 nm and at 420 nm corresponding to intermediate- and high-molecular-weight products [13]. The results indicate that the absorbance values at 345 nm and 420 nm in G and GI fractions were higher in SBM samples than in CBM. However, for the colonic fermentation fraction, the absorbance values at 345 nm and 420 nm were significantly higher in CBM than in SBM. Furthermore, the highest amount of premelanoidins and melanoidins for both samples (CBM and SBM) was obtained in the F fraction. This finding is in accordance with previous reports [9,16] confirming the important role of colonic fermentation in the bioaccessibility of melanoidins.

The bioactivity of the bioaccessible melanoidins was evaluated by assessing their antioxidant capacity (Q-ABTS and Q-FRAP) and radical scavenger capacity (Q-SRSC and Q-HRSC) (Table 1). When comparing CBM and SBM bioaccessible melanoidins, the antioxidant and radical scavenger capacities were found to be dependent on the method. The results show that both samples had similar antioxidant capacities evaluated using Q-ABTS and a similar capacity to inhibit the hydroxyl radical (Q-HRSC). However, significant differences were observed in the reducing capacity evaluated through the Q-FRAP method, where the SBM showed a higher reducing capacity in the GI and CF fractions but lower reducing capacity in the G fraction. The CBM and SBM bioaccessible samples from the different digestion stages exhibited significant changes in antioxidant and radical scavenger capacity throughout the process of in vitro gastro-intestinal digestion. In general, the antioxidant and radical scavenger activities were lower in the gastric fraction and higher in the GI fraction, except for Q-FRAP. These results indicate that the intestinal digestion of CBM and SBM extracts mainly contributes to the antioxidant capacity of the bioaccessible fractions. Additionally, the F fraction showed important antioxidant and radical scavenger activity, which can be attributed to the release of microbial metabolites with high antioxidant and radical scavenger activity during melanoidin fermentation [9,23,24].

The chelating capacity of melanoidins is of high importance as it offers protection against oxidative stress. Transition metals are one of the most important pathways for oxidation, which produces reactive oxygen species, mainly hydroxyl radicals, that oxidize biomolecules in cells. Melanoidins act as anionic hydrophilic polymers that can bind transition metals such as iron to form chelates and prevent or delay the production of free radicals [5,25]. According to our results, the metal chelating capacity of bioaccessible melanoidin fractions importantly increased with colonic fermentation. This could be attributed to the presence of compounds, such as carbohydrate, in this fraction, which is an important parameter for melanoidin-iron chelation [20,26,27]. The significantly higher chelating activity for the G and F bioaccessible fractions of CBM compared to SBM indicates a relationship between melanoidin activity and food bioaccessibility. This activity in the bioaccessible fractions is important to prevent iron absorption, prooxidant activity, and cytotoxic effects [5,28].

**Figure 1:**
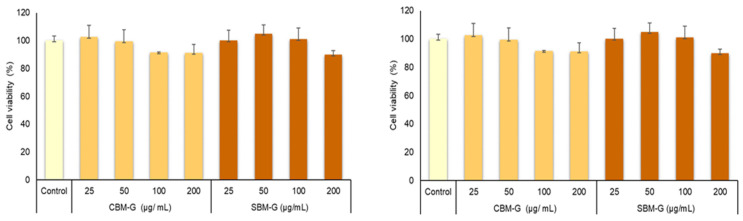

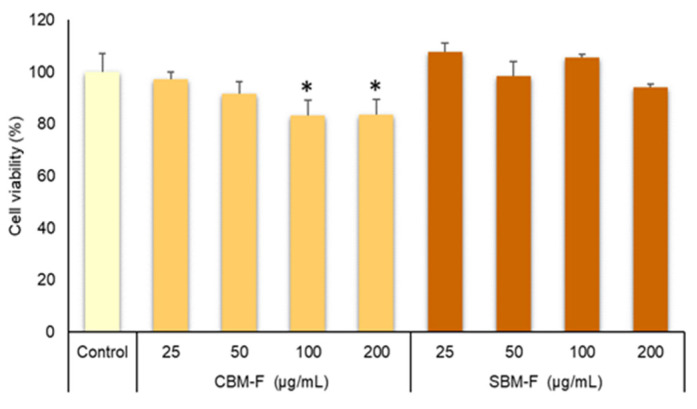
Cell viability of Caco-2 cell treated with CBM and SBM bioaccessible fractions assessed using MTT assay. The results are expressed as a percentage of viability with respect to the control cells (C) and are expressed as mean ± standard deviation (n = 4). Asterisk (*) refers to significant differences, *p* < 0.05, between the digestion fractions and the control cells. CBM: common bread melanoidins; SBM: soft bread melanoidins. Gastric (G), gastrointestinal (GI), and colonic fermentation (F) fractions of CMB and SBM samples.

Although, in a previous study, we observed that crust bioaccessible melanoidins obtained through in vitro digestion using Pronase E and bioaccessible melanoidins achieved by a complete in vitro gastrointestinal digestion did not show cytotoxicity at different concentrations in both Caco-2 and HUVEC cells [3], the effects of the different digestion fraction (gastric and gastrointestinal) and colonic fermentation were not analyzed. Therefore, in this study, we evaluate the effects of the bioaccessible melanoidin fractions on the cell viability using two strategies: endpoint evaluation using MTT assay and real-time cellular analysis (RTCA) by measuring the cellular growth curves. The viability percentages of Caco-2 cells determined by MTT assays (Figure 1) were higher than 80% after 24 h of treatment with all digestion fractions (G, GI, and F) at concentrations of 25, 50, 100, and 200 μg/mL Therefore, these results indicate no cytotoxicity of the different melanoidin bioaccessible fractions and confirm the observations of other authors who did not observe cytotoxic effects provided by bread crust melanoidins in Caco-2 models using the MTT assay [3,29].

**Figure 2:**
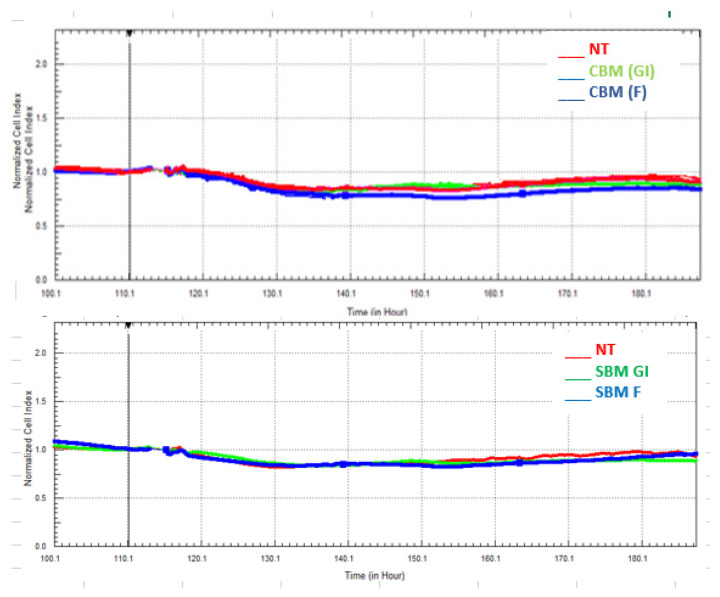

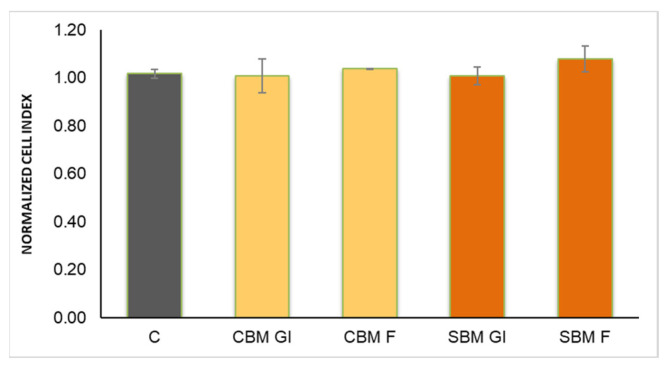
Real-time monitoring of bioaccessible fractions mediating cell viability and proliferation. A period of 24 h after seeding, Caco-2 cells were treated with 100 μg/mL of the gastrointestinal (GI) and colonic (F) fractions of common (CBM) and soft (SBM) bread melanoidins, and cell index was continuously monitored for a total of 180 h. The figures represent the growing curves under the treatment with the bioaccessible fractions CBM and SBM with time. The bar graph represents the normalized cell index for the bioaccessible samples after 4 h of incubation with the digestion fractions. Results are expressed as mean values and standard deviation (n = 3).

The other method used to evaluate the cytotoxicity of the bioaccessible melanoidins was real-time cellular analysis (RTCA). This study was conducted by measuring the changes in electrical impedance using the xCELLigence system (Figure 2). The system detects the loss of barrier function involved in the development of intestinal diseases [18]. It is well known that a decrease in impedance corresponds to an increase in intestinal permeability, and hence the evaluation of intestinal cell impedance represents an appropriate method for assessing the potential cytotoxicity of bioaccessible compounds. Our results (Figure 2) indicate that the integrity of the Caco-2 cell monolayer was not affected by the treatment with GI and F digestion-fractions as reflected by the absence of changes in impedance values (Figure 2) over time of treatment. Cell index values did not show significant differences compared to untreated cells. These results were observed for the GI and F digestion-fractions of both types of melanoidins (CBM and SBM).

**Figure 3:**
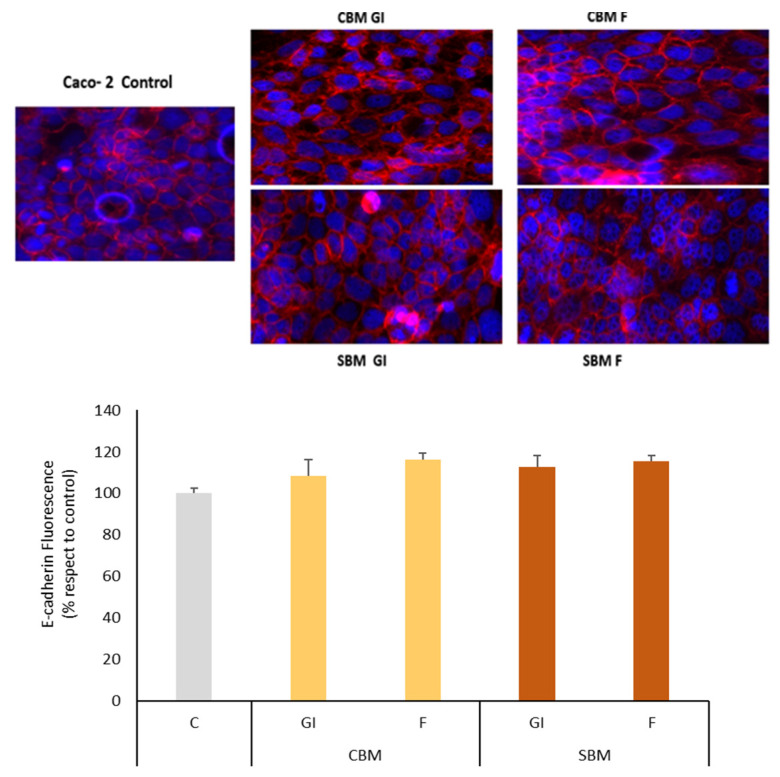
E-cadherin of Caco-2 cells of cells treated with bread melanoidins digested fractions assessed by immunofluorescence. Differenced Caco-2 cells were incubated with 100 μg/mL of gastrointestinal (GI) or colonic fermentation (F) fractions of common bread melanoidins (CBM) or soft bread melanoidins (SBM). C = control cells. Results are expressed as mean values and standard deviation (n = 3). Results are expressed as mean values ± standard deviation (n = 3).

In addition, we evaluated the effect of bioaccessible melanoidin fractions on E-cadherin, an adherens-junction protein that maintains membrane integrity and is a vital component of the Caco-2 cell intestinal barrier. E-cadherin was analyzed by immunofluorescence in Caco-2 cell monolayers incubated without and with 100 g/mL of digestion-fractions of melanoidins for 4 h (Figure 3). No changes in E-cadherin distribution were observed after treatment, thus confirming the results that GI and CF fractions of melanoidins do not affect membrane integrity.

**Figure 4:**
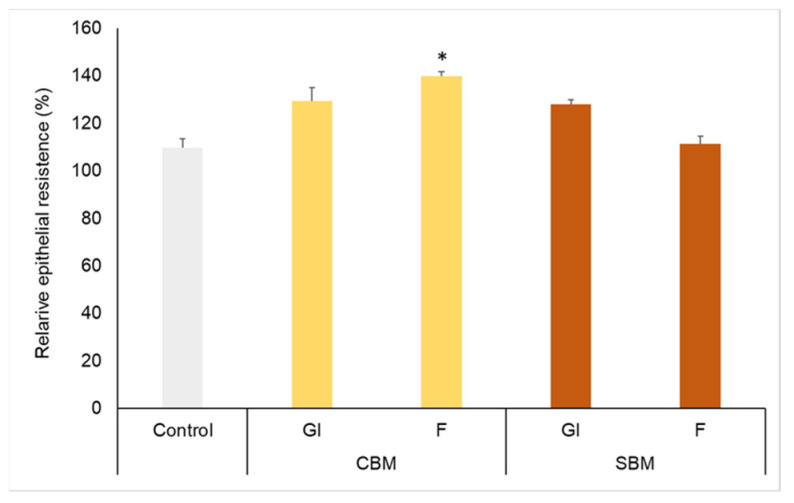
TEER values from Caco-2 cells treated with CBM and SBM bioaccessible melanoidins. Relative epithelial resistance of Caco-2 cells treated with melanoidins. GI: Caco cells treated with gastrointestinal fractions; F: Caco cells treated with colonic fraction. CBM: common bread melanoidins. SBM: soft bread melanoidins. Results are expressed as mean ± SD (n = 3). Asterisk (*) refers to significant differences; *p* < 0.05, between the digestion fractions and the control cells.

The bioactivity of melanoidins also depends on their transport through the intestinal barrier. This can be a limiting factor if these fractions are not able to cross the barrier of epithelial cells that line the intestinal wall. To investigate the effect of GI and F digestion fractions on the transport of melanoidins across intestinal cells, the human adenocarcinoma cell line Caco-2 was used. Caco-2 cells are capable of forming a monolayer and differentiating, acquiring functions similar to those of intestinal epithelial cells. Melanoidins from the gastrointestinal (GI) and colonic fermentation (F) fractions were deposited in the apical part of the transwell support containing the monolayer of differentiated cells. The integrity of the monolayer before and after the treatment with bioaccessible melanoidins was evaluated using trans-epithelial electrical resistance (TEER) values (Figure 4). The change in TEER values in both control and treated cells was lower than 20% with respect to the pretreatment values, and a decrease in relative epithelial resistance was not observed between control cells and those treated with the different GI or F digestion-fractions. Therefore, the integrity of the monolayer was not affected by treatment with bioaccessible melanoidins (GI and CF). Several studies indicate the importance of bioactive food components (polyphenols, MRPS) in maintaining or increasing TEER to protect against a loss in intestinal permeability [30,31,32].

**Figure 5:**
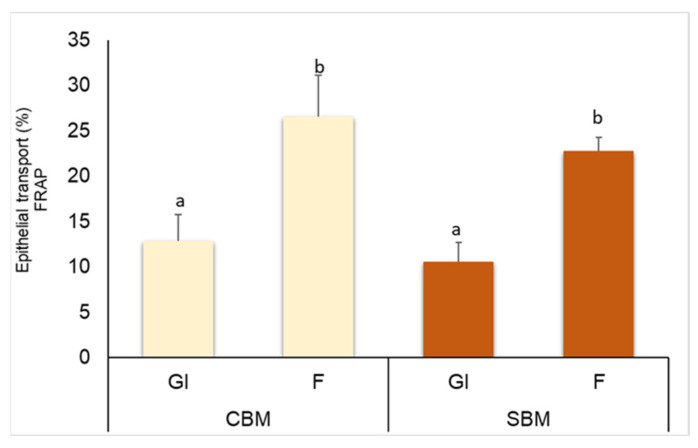
Antioxidant capacity measured as FRAP capacity of bioavailable fractions evaluated as epithelial transport (%). Results are shown as mean ± SD (n = 3), and statistics were represented by performing a one-way ANOVA, in which samples with different letters indicate significant differences (*p* < 0.05). CBM: common bread melanoidins; SBM: soft bread melanoidins. GI: gastrointestinal digestion fraction; F: colonic fermentation fraction.

Furthermore, the transport of melanoidins through the monolayer was confirmed by evaluating their antioxidant capacity. If the compounds are able to cross the cellular barrier, then the antioxidant capacity of the basolateral fraction must be increased (Figure 5). Therefore, the antioxidant capacity, measured using the FRAP method, of the apical and basolateral fractions was evaluated, and the result is represented as percentage (%) of epithelial transport (%). FRAP activity (Figure 5) was significantly higher in the colonic fermentation fractions of both CBM and SBM compared with the GI fraction. Other authors have pointed out that the fraction of melanoidins that crosses through the intestinal barrier still presents antioxidant capacity, in addition of being able to act at the cellular level as an antioxidant defense against the production of reactive oxygen species [33]. Therefore, we confirmed that GI and F bioaccessible melanoidins that have crossed the intestinal barrier remain with a high antioxidant activity that can provide beneficial effects at the cellular level.

**Figure 6:**
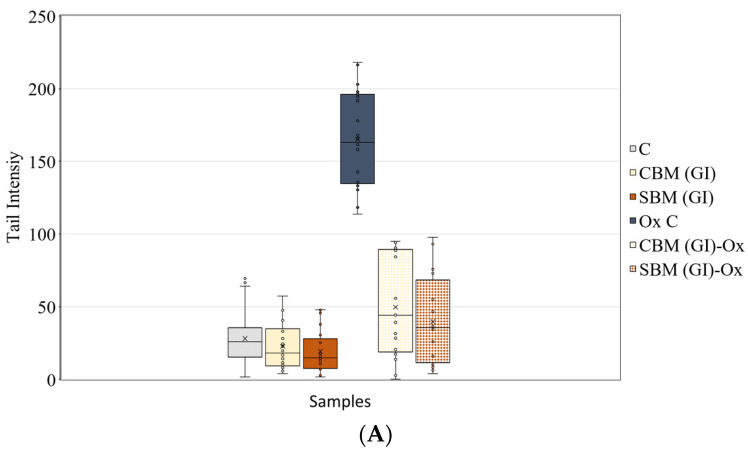

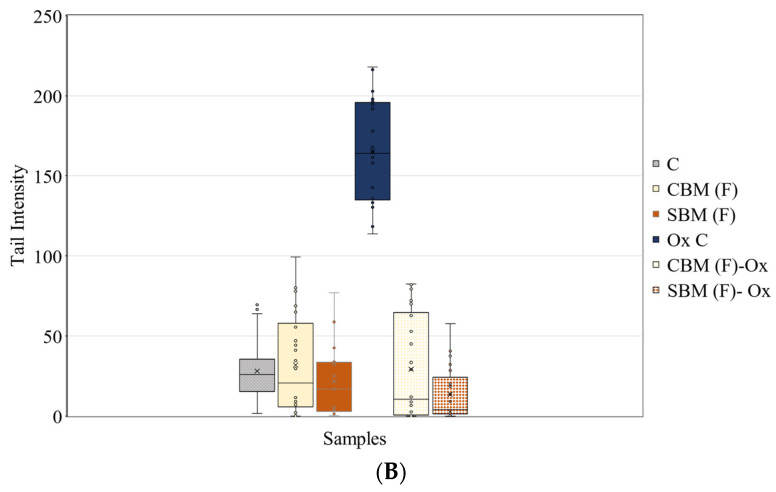
Antigenotoxic effects of bioaccessible fractions of melanoidins in Caco-2 cell. (**A**) C: control cells; CBM (GI): cells incubated in presence of 100 μg/mL of GI fraction of common bread melanoidins; SBM (GI): cells incubated in presence of 100 μg/mL of GI fraction of soft bread melanoidins; OxC: Oxidized control, cells treated with 0.5 mM of H_2_O_2_. CBM(GI)-Ox: cell preincubated with 100 μg/mL of GI-CBM and treated with 0.5 mM of H_2_O_2_; SBM(GI)-Ox: cell preincubated with 100 μg/mL of GI-SBM and treated with 0.5 mM of H_2_O_2_; (**B**) C: control cells; CBM (F): cells incubated in presence of 100 μg/mL of F fraction of common bread melanoidins; SBM (F): cells incubated in presence of 100 μg/mL with F fraction of soft bread melanoidins; OxC: Oxidized control, cells treated with 0.5 mM of H_2_O_2_; CBM(F)-Ox: cell preincubated with 100 μg/mL of F-CBM and treated with 0.5 mM of H_2_O_2_; SBM(F)-Ox: cell preincubated with 100 μg/mL of F-SBM and treated with 0.5 mM of H_2_O_2_. The results are the values of DNA migration evaluated by the comet tail length (n = 30 cells for each sample) and are expressed as content tail intensity (DNA damaged).

When considering the genoprotective effect of melanoidins, it becomes essential to know the safety of these compounds before utilizing them as functional ingredients. Other studies involved model melanoidins (glucose/glycine) that have been evaluated using methods such as the Ames test or micronucleus test where it was observed that melanoidins did not exhibit genotoxicity [34]. To assess the genoprotective effect of melanoidins, it is necessary to ensure the safety of these compounds in bioaccessible fractions. Therefore, we evaluated the genoprotective effect of GI and CF samples in the Caco-2 cell line using the comet assay. The results of this study are presented in Figure 6.

Treatment of Caco-2 cells with bioaccessible fractions (GI and F) of both samples (CBM and SBM) did not induce DNA damage and showed no significant differences compared to the control. The treatment with H_2_O_2_ (our positive control of genotoxic damage) induced an increase in tail intensity, indicating high DNA damage. The pretreatment of oxidized cells with the bioaccessible fractions resulted in a high genoprotective effect for all bioaccessible fractions. The most remarkable finding was that the bioaccessible fractions (GI and F) from SBM samples provided the highest degree of DNA oxidation protection. Similar results were observed by our research group who employed the comet assay to evaluate the genoprotective effect in the fractions obtained from gastrointestinal digestion of instant coffee melanoidins [19]. These results suggest that the different structure of CBM and SBM melanoidins results in bioaccessible fractions with different genoprotective effects.

## 4. Conclusions

Both common and soft bread crust melanoidin extracts exhibited high bioaccessibility, which was influenced by the type of bread crust and the digestion phase, with higher values observed in the colonic fermentation fraction. This fraction showed a significant increase in melanoidin content compared to the gastrointestinal fraction. The bioaccessible melanoidins exhibited an antioxidant capacity, chelating capacity, and radical scavenger activity that were dependent on the evaluation method used. Furthermore, our study confirmed the absence of cytotoxic effects of bioaccessible melanoidins on Caco-2 cells, as assessed by the MTT assay and real-time impedance measurement. Treatment with bioaccessible fractions did not affect the distribution of E-cadherin in Caco-2 cells, confirming their safety in the maintenance of epithelial barrier integrity. Moreover, our study not only demonstrated the transport of melanoidins across the intestinal barrier without changes in cell permeability but also that melanoidins exhibited an antioxidant activity on the basolateral side of the cell monolayer and a genoprotective effect under oxidative stress conditions.

Overall, our findings highlight and emphasize the importance of bioaccessible fractions obtained through gastrointestinal digestion and colonic fermentation in evaluating the biological efficacy of melanoidins. These insights contribute to a better understanding of the bioaccessibility and bioactivity of crust bread melanoidins, opening avenues for future applications in functional food development. Further studies are needed to characterize the structure of the bioaccessible fraction of crust bread melanoidins and establish a relationship with the health effects of these compounds.

## Figures and Tables

**Table 1:** Characterization of bioaccessible melanoidins (gastric, gastrointestinal, and colonic fermentation) of common bread and soft bread crust.

	COMMON BREAD (CBM)	SOFT BREAD (SBM)
**ABSORBANCE (UA)**		
**Gastric (G) (345 nm)** **Gastrointestinal (GI) (345 nm)** **Colonic fermentation (F) (345 nm)** **Gastric (G) (420 nm)** **Gastrointestinal (GI) (420 nm)** **Colonic fermentation(CF) (420 nm)**	0.383 ± 0.013 ^b^ 0.270 ± 0.007 ^a^ 0.437 ± 0.013 ^c^ 0.131 ± 0.004 ^a^ 0.141 ± 0.005 ^a^ 0.189 ± 0.007 ^b^	0.397 ± 0.005 ^b^ 0.286 ± 0.001 ^a,^* 0.389 ± 0.008 ^b,^* 0.179 ± 0.002 ^c,^* 0.142 ± 0.005 ^a^ 0.163 ± 0.004 ^b,^*
**TOTAL ANTIOXIDANT CAPACITY (TAC)**		
**Q-ABTS (µG TROLOX/mg)**		
**Gastric (G)** **Gastrointestinal (GI)** **Colonic fermentation(F)**	1.47 ± 0.02 ^a^ 4.35 ± 0.06 ^b^ 4.45± 0.01 ^c^	1.40 ± 0.14 ^a^ 4.29 ± 0.04 ^b^ 4.40 ± 0.02 ^b^
**Q-FRAP (µG FE (II)E/mg)**		
**Gastric (G)** **Gastrointestinal (GI)** **Colonic fermentation(F)**	3.66 ± 0.05 ^c^ 0.94 ± 0.15 ^a^ 1.94 ± 0.06 ^b^	3.08 ± 0.07 ^c,^* 1.42 ± 0.01 ^a,^* 2.54 ± 0.03 ^b,^*
**Q-SRSC (% Inhibition)**		
**Gastric (G)** **Gastrointestinal (GI)** **Colonic fermentation(F)**	54.9 ± 8.0 ^a^ 84.3 ± 5.8 ^c^ 61.7 ± 1.2 ^b^	47.6 ± 8.6 ^a,^* 82.9 ± 8.5 ^c^ 76.6 ± 5.6 ^b,^*
**Q-HRSC (%Inhibition)**		
**Gastric (G)** **Gastrointestinal (GI)** **Colonic fermentation(F)**	38.7 ± 7.5 ^a^ 83.9 ± 6.2 ^c^ 72.6 ± 0.67 ^b^	33.2 ± 2.33 ^a^ 84.8 ± 4.28 ^c^ 73.2 ± 0.98 ^b^
**CHELATING CAPACITY (%)**		
**Gastric (G)** **Gastrointestinal (GI)** **Colonic fermentation(F)**	3.82 ± 0.25 ^a^ 4.23 ± 0.24 ^a^ 72.2 ± 1.45 ^b^	1.74 ± 0.64 ^a,^* 4.77 ±0.76 ^b^ 62.9 ± 1.85 ^c,^*

Data expressed as mean values ± standard deviation (n = 4). Means in a row with different letters are significantly different (*p* < 0.05). Asterisk (*) refers to significant differences, *p* < 0.05, between CBM and SBM samples. TE: Trolox equivalents. Fe (II) E: iron (II) equivalents.

## Data Availability

Data from the present study are available upon request from the corresponding author. The availability of the data is restricted to investigators based in academic institutions.
